# Antimicrobial effects of liquid anesthetic isoflurane on *Candida albicans*

**DOI:** 10.1186/1479-5876-4-46

**Published:** 2006-11-09

**Authors:** Viachaslau M Barodka, Edward Acheampong, Garry Powell, Ludmila Lobach, David A Logan, Zahida Parveen, Valerie Armstead, Muhammad Mukhtar

**Affiliations:** 1 Anesthesiology Program For Translational Research, Department of Anesthesiology, Thomas Jefferson University, Philadelphia, Pennsylvania, USA; 2 Division of Infectious Diseases, Department of Medicine, Thomas Jefferson University, Philadelphia, Pennsylvania, USA; 3 Department of Biological Sciences, Clark Atlanta University, Atlanta, Georgia, USA; 4 Department of Microbiology and Immunology, Institute for Molecular Medicine and Infectious Disease, Drexel University College of Medicine, Philadelphia, Pennsylvania, USA

## Abstract

*Candida albicans *is a dimorphic fungus that can grow in yeast morphology or hyphal form depending on the surrounding environment. This ubiquitous fungus is present in skin and mucus membranes as a potential pathogen that under opportunistic conditions causes a series of systemic and superficial infections known as candidiasis, moniliasis or simply candidiasis. There has been a steady increase in the prevalence of candidiasis that is expressed in more virulent forms of infection. Although candidiasis is commonly manifested as mucocutaneous disease, life-threatening systemic invasion by this fungus can occur in every part of the body. The severity of candidal infections is associated with its morphological shift such that the hyphal morphology of the fungus is most invasive. Of importance, aberrant multiplication of *Candida *yeast is also associated with the pathogenesis of certain mucosal diseases. In this study, we assessed the anti-candidal activity of the volatile anesthetic isoflurane in liquid form in comparison with the anti-fungal agent amphotericin B in an *in vitro *culture system. Exposure of *C. albicans *to isoflurane (0.3% volume/volume and above) inhibited multiplication of yeast as well as formation of hyphae. These data suggest development of potential topical application of isoflurane for controlling a series of cutaneous and genital infections associated with this fungus. Elucidiation of the mechanism by which isoflurane effects fungal growth could offer therapeutic potential for certain systemic fungal infections.

## Background

*Candida albicans *is an opportunistic fungal pathogen mainly causing infections among immunocompromised individuals [1]. Infections associated with this fungus range from superficial mycoses to life threatening systemic candidiasis, which involve various body organs and invasive mucosal disorders. A wide spectrum of infections associated with *C. albicans *are ascribed to the capability of this fungus to switch between unicellular budding yeast to multicellular, filamentous mycelial or hyphal form [2]. Several studies in the past have reported that the hyphal form of *C. albicans *is more invasive compared with budding yeast [2-4]. This hypothesis has been further strengthened in a rodent model of fungal infection where *C. albicans *strains unable to form hyphae were essentially avirulent [5]. As such, there has always been a need for an antifungal agent that can both prevent the formation of hyphae as well as destroy both yeast and hyphal morphologies of this fungus.

Isoflurane is a halogenated volatile anesthetic and its gaseous form has shown antibacterial activity against one of the most common bacterial pathogens in hospital-acquired pneumonia *Pseudomonas aeruginosa *[6]. One study on the other hand did not find any antibacterial activity of isoflurane against *Staphylococcus aureus *and *Escherichia coli *[7]. Prospects for clinical usage of the gaseous isoflurane as an antibacterial agent have been hampered due to adverse systemic effects and occupational hazards [8,9]. Encouraged by our initial data showing that the gaseous form of isoflurane inhibits *C. albicans *morphogenesis (formation of hyphae from yeast cells, unpublished data), we analyzed the anti-candidal potential of volatile liquid isoflurane. *C. albicans in vitro *cultures were treated with various concentrations of isoflurane in semi-anaerobic as well as aerobic environments. Growth behavior and morphological changes were then assessed. For our initial studies, semi-anaerobic study design was essentially based on the volatile nature of the isoflurane with the challenge to maintain uniform concentrations of volatile anesthetic in the *in vitro *culture. Moreover, like several other fungi, *C. albicans *is a facultative anaerobe and can fulfill energy requirements either in the presence of oxygen or in the absence via fermentation [10]. Intriguingly, aerobic cultures of the yeast treated with isoflurane also revealed inhibitory effects on both yeast multiplication and formation of hyphae. We observed that isoflurane could completely abolish formation of hyphae in *C. albicans*, a step essential for the pathogenesis of this fungus *in vivo*. A concentration of 0.5% isoflurane (v/v) showed fungicidal activity equivalent to amphotericin B, 4.0 μg/ml a concentration that has been previously reported as fungicidal [11]. Moreover, *C. albicans *treated with lower concentrations of isoflurane (0.1 – 0.4%) showed diminished growth. These findings are highly significant, as this approach will provide a platform for a systematic study of fungal morphogenesis at the molecular level. Moreover, isoflurane in gaseous form is a widely used anesthetic approved by the U.S. Food and Drug Administration (FDA). Although usage of the gaseous form of isoflurane to treat systemic infections may not be feasible due to its anesthetic actions as well as adverse cardiovascular effects at high concentrations, however, possibilities exist for manipulation of the antimicrobial potentials of isoflurane. This is the first report revealing liquid isoflurane-mediated control of *C. albicans *growth and morphogenesis in a concentration-dependent manner. These findings suggest that there may be an opportunity to develop topical microbicides based on creative formulations that modulate the release of isoflurane. This strategy may provide a means for controlling *Candida *and related superficial infections that affect millions of immunocompromised and healthy individuals worldwide.

## Results

### Effects of liquid isoflurane on the metabolic activity of *C. albicans*

In our pilot studies we evaluated the effects of clinically relevant concentrations of gaseous isoflurane on *C. albicans *morphogenesis and observed an inhibitory effect on the formation of true hyphae from the yeast form of this fungus (data not illustrated). Encouraged by our initial observations we further analyzed the effects of various concentrations of liquid isoflurane on the morphogenesis and growth of *C. albicans*.

**Figure 1:**
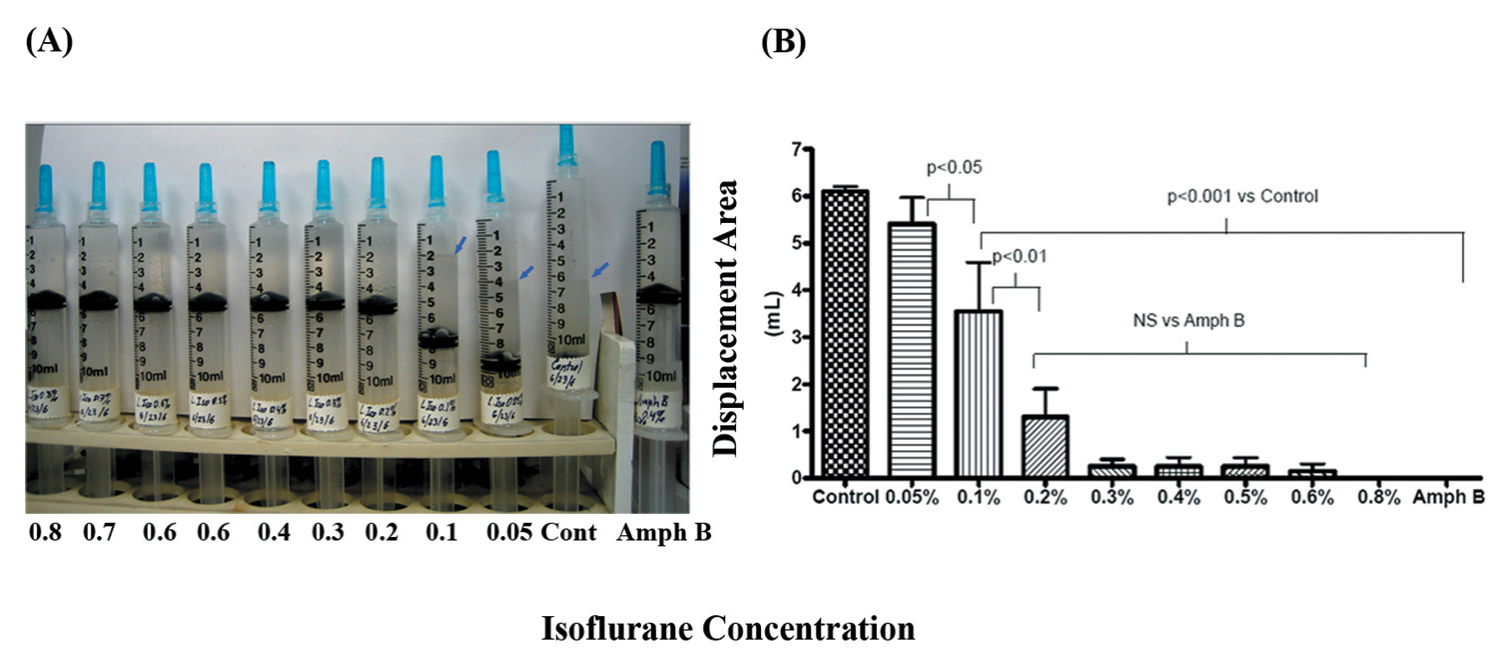
**Effects of various concentrations of isoflurane on metabolic activity of candidal yeast culture**. Actively growing yeast cultures were treated with various concentrations of isoflurane (0.05% 0.8% v/v of media) and amphotericin B (4 μg/ml). The top of each syringe was tightly sealed to create semi-anaerobic environment. The metabolic activity of each syringe was determined by volume of displacement (an indicator of metabolic activity of Candidal yeast) in the syringe. Panel A shows volume of displacement in syringes (Smaller arrows indicate the levels) whereas Panel B is graphic representation of the displaced volume. Columns in the graph represent the means (+/- Standard error of the mean (SEM)) of four independent experiments.

Isoflurane concentrations of 0.1% (v/v) and higher significantly inhibited metabolic activity of *C. albicans *(p < 0.001 compared to control), however, a concentration of 0.4–0.5% (v/v) was critical in this study to achieve inhibitory activity similar to that of amphotericin B (4.0 μg.mL) (Fig 1B). Concentrations of liquid isoflurane less than 0.1% (v/v) did not significantly inhibit metabolic activity (determined by the fungi-derived gas volume displacement in syringe culture) of *C. albicans *in a semi-anaerobic environment. (Fig. 1A).

### Effect of isoflurane on candidal growth and multiplication

**Figure 2:**
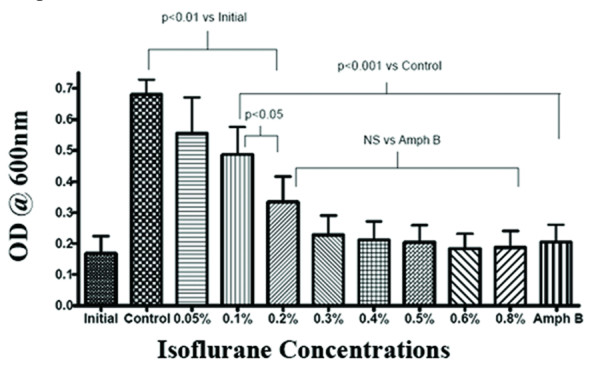
**Effects of isoflurane on growth of *Candida albicans *in semi-anaerobic environment**. The effect of various concentrations of isoflurane and anti-fungal Amphotericin B 4 μg/ml (Amph B) on the growth of syringe culture was determined by optical density (OD) reading at 600 nm. Initial is the OD of starter culture. Columns represent the means (+/-) Standard error of the mean (SEM)) of four independent experiments. NS = Not significant

**Figure 3:**
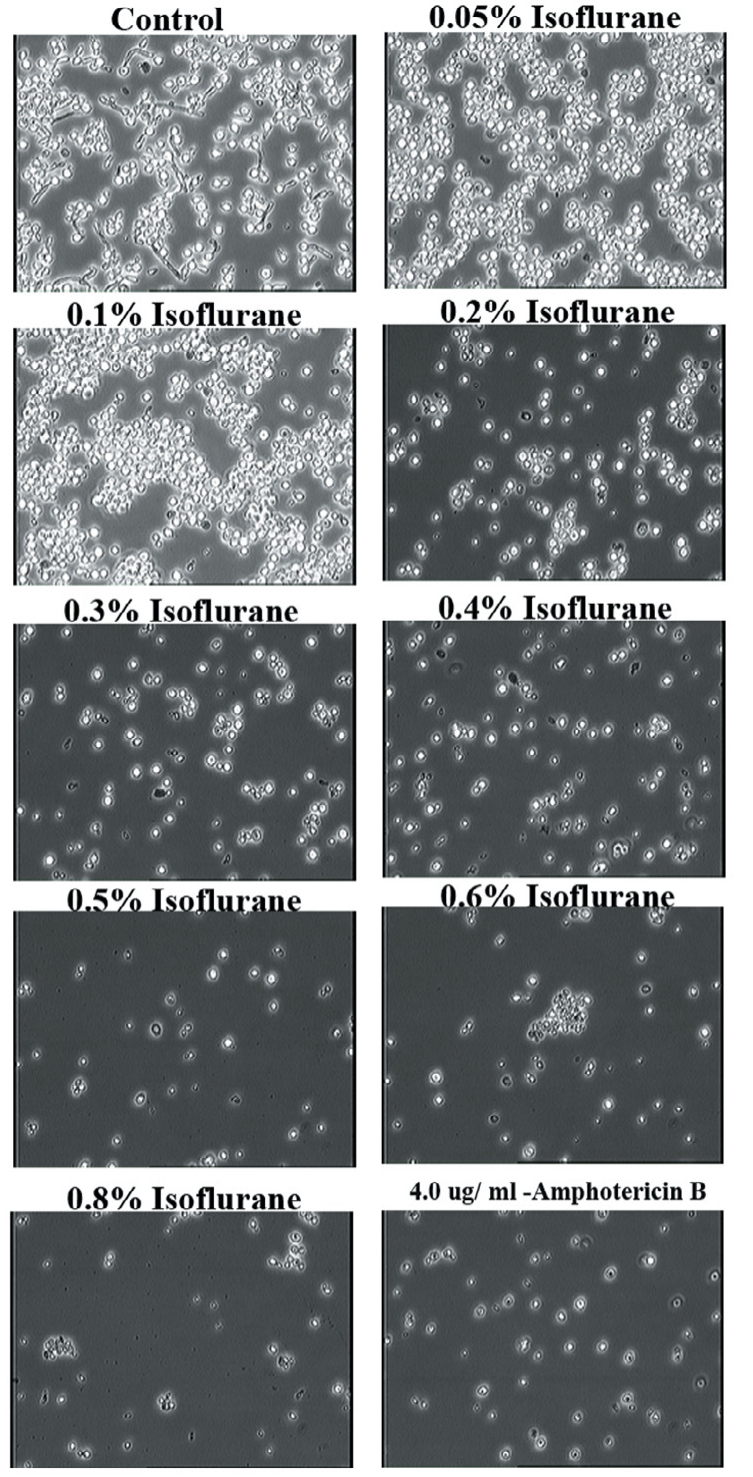
**Effects of various concentrations of isoflurane and anti-fungal amphotericin B**. Morphological changes in *Candida albicans *treated with various concentrations of isoflurane and amphotericin B in semi-anaerobic conditions. The magnification of each picture is 60×. The concentrations of isoflurane and amphotericin B are indicated in each panel. Data presented in this figure is representative of four independent experiments.

Isoflurane concentrations of 0.1% (v/v) and above were inhibitory to yeast multiplication with concentrations of 0.2% or higher having an effect similar to amphotericin B in a semi-anaerobic environment (Fig. 2). Furthermore, even at the lowest concentration (0.05%) liquid isoflurane completely inhibited the formation of hyphae in semi-anaerobic conditions (Fig. 3).

**Figure 4:**
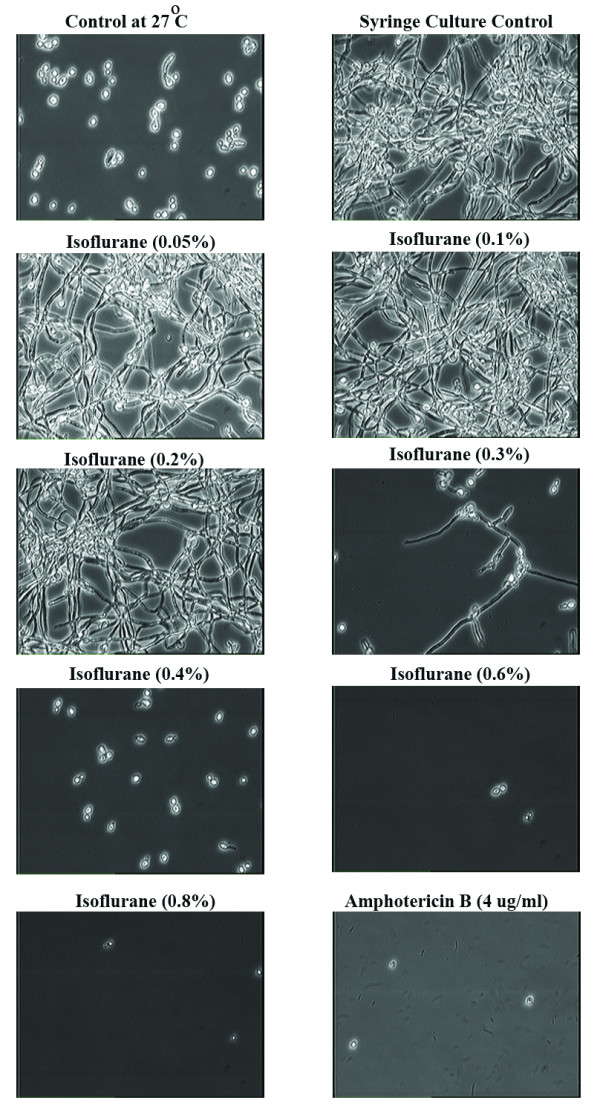
**Viability Assay of *Candida albicans *culture treated with Isoflurane in semi-anaerobic environment**. For determination of viability assays 200 μl fungal culture from each syringe was added to 5 ml of prewarmed Lee's medium and allowed to grow at 37°C for 16 hrs. Photomicrographs of each fungal culture were captured by taking 100 μl of fungal culture on glass slides and observing under the microscope. Data presented in this figure is representative of three independent experiments.

Although lower concentrations (0.1 – 0.3%) of isoflurane revealed inhibitory effects on growth, viability assays of the cultures treated with these concentrations indicated that the yeast were capable of forming hyphae equivalent to control cultures (Figure 4). However, at isoflurane concentrations of 0.3% and above we observed a significant inhibitory effect on the formation of hyphae. When compared with positive control amphotericin B, a well-characterized and clinically used antifungal agent, a concentration of 0.4% isoflurane is equivalent to 4 μg/ml of amphotericin B which is a fungicidal concentration [11].

**Figure 5:**
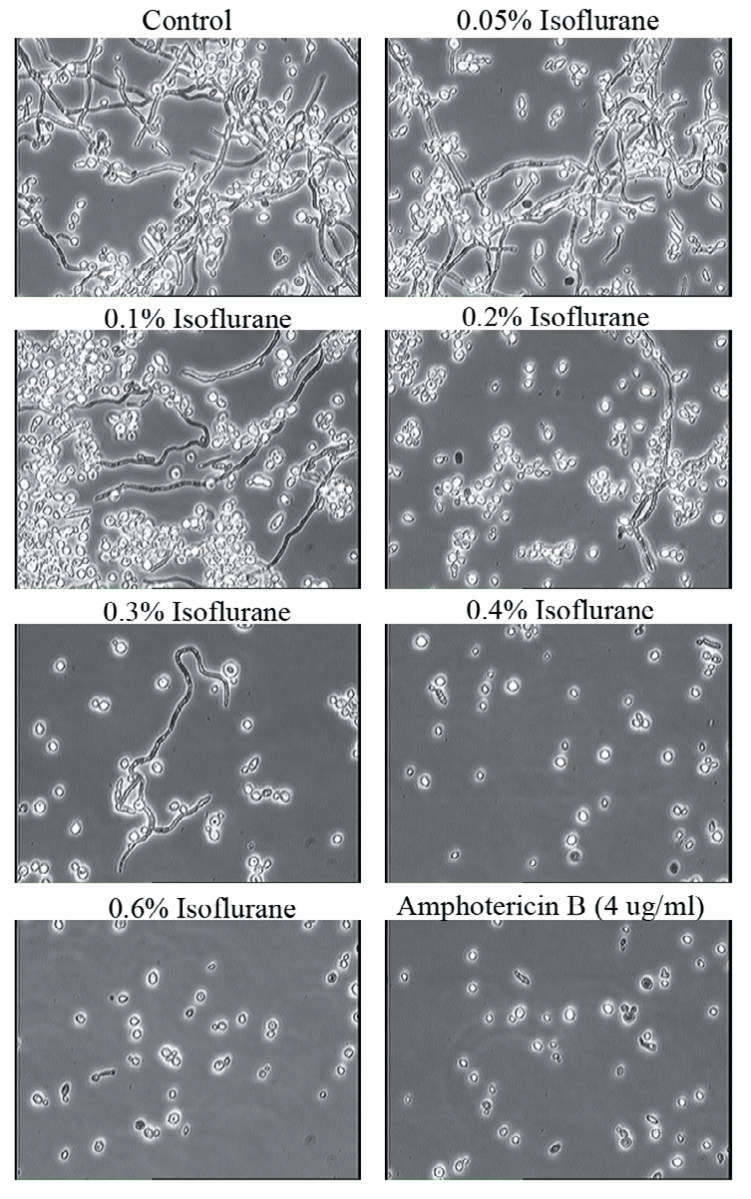
**Morphological changes in *Candida albicans *treated with various concentrations of isoflurane and amphotericin B in aerobic conditions**. The effect of various concentrations of isoflurane and anti-fungal Amphotericin B was determined microscopically. Each culture was thoroughly mixed and 100 μl from each syringe was spread on microscopic slides to capture images at (60×). Data presented in this figure is representative of three independent experiments.

**Figure 6:**
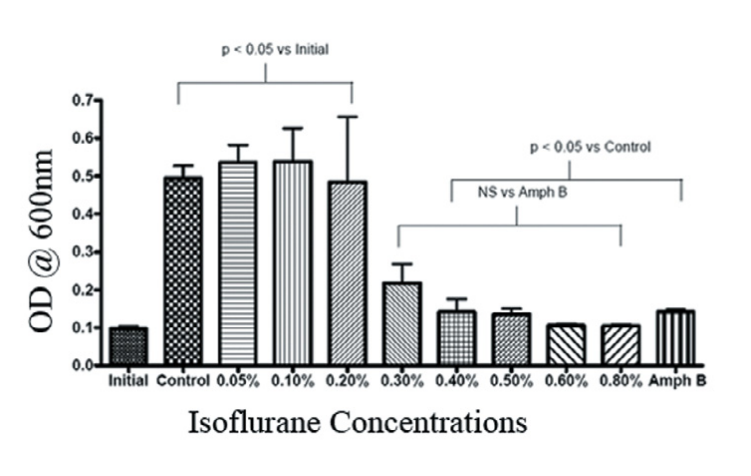
**Effects of isoflurane on growth of *Candida albicans *in aerobic environment**. The effect of various concentrations of isoflurane and anti-fungal Amphotericin B on the growth of syringe culture was determined by OD reading at 600 nm. The data presented in this figure is an average of three independent experiments. Columns in the graph represent the means (+/-) Standard error of the mean (SEM).

To further discern whether liquid isoflurane has similar effects on *C. albicans *growth and morphogenesis under an aerobic environment, *Candida *yeast cultures treated with various concentrations of liquid isoflurane were allowed to grow at 37°C with the syringe caps loosened (completely aerobic environment). The result was that growth of Candida yeast was significantly inhibited at 0.2–0.3% (Figure 5); however, lower concentrations of isoflurane did not show an inhibitory effect on the formation of hyphae as observed under semi-anaerobic conditions. However, higher concentrations of 0.4% and above were capable of inhibiting yeast multiplication as well as formation of hyphae as depicted in Figure 6 as well.

However, the non-isoflurane-treated control culture under semi-anaerobic conditions showed the ability to form small hyphae after 16 hrs as shown in Figure 3 (Control). The growth inhibitory effect of 0.4% isoflurane was equivalent to 4.0 μg/ml of amphotericin B. Both isoflurane and amphotericin B inhibited formation of hyphae in *Candida*, which is the form that is commonly associated with pathogenicity.

An interesting observation pertains to the cellular volume of yeast cells. Shrinkage in cellular volume of the yeast was obvious from cultures treated with isoflurane at a concentration of 0.2% (Fig. 3). The number of shrunken cells increased with the higher concentrations of isoflurane employed in these experiments. Amphotericin B as described previously also showed cellular shrinkage in our studies [12,13].

## Discussion

*Candida albicans *is a dimorphic opportunistic pathogen causing both superficial and systemic infections. The most prominent factors involved in the pathogenesis of this fungus are: capability to exist as both yeast and mycelium with filamentous hyphae (dimorphism) depending on host environment [14]. The hyphal form of this fungus is more invasive and is also involved in the secretion of various proteases and lipases which facilitate tissue invasion of the fungus [2]. Moreover, phenotypic switching between hyphae and yeast form has been proposed as a regulatory element in the pathogenesis of this fungus [15]. In the past, efforts have been directed to identify molecular mechanisms of phenotypic switching between yeast and hyphal morphology of this fungus [16-18].

Like other fungi, an interesting aspect of *Candida *is its facultative nature [19] and as such can fulfill its energy requirement either by aerobic respiration or anaerobic fermentation. In our studies we determined antifungal activity of liquid isoflurane in both aerobic and semi-anaerobic environment. Semi-anaerobic environment was created due to the volatile nature of isoflurane, however, in our studies we also used an air-tight chamber and exposed Candida cells with previously described, clinically relevant concentrations of gaseous of isoflurane [1–3 minimum alveolar concentration (MAC), a term used by anesthetists for defining the strength of anesthetic vapors] that inhibited formation of true hyphae from the yeast (Data not illustrated in this manuscript). Moreover, previous reports suggest that the sensitivity of *C. albicans *to antifungal drugs particularly Terbinafine, neticonazole and amphotericin B do not vary between aerobic and anaerobic environments [20].

Interestingly, liquid isoflurane treatments under aerobic conditions inhibited multiplication of yeast in the same manner as semi-anaerobic environments. However, in aerobic conditions, formation of hyphae were observed in fungal yeast treated with lower concentrations of isoflurane (0.05 – 0.2%) in contrast to semi-anaerobic. It is not clear if this phenotypic difference is due to nuances in toxicity of isoflurane and or different membrane conditions. Despite these differences, higher concentrations of isoflurane showed similar effects under both semi-aerobic and aerobic environments.

As far as growth conditions are concerned, there are few studies describing fungal growth in anaerobic environments in spite of the facultative nature of this fungus. Efforts have been directed towards understanding the survival of *C. albicans *in anaerobic environments [21,22]. One study describes anaerobic morphogenesis of this fungus in synthetic medium supplemented with yeast extract or a combination of oleic acid, nicotinic acid and ammonium chloride [23]. Although our findings are quite contrary to this study, i.e. limited formation of hyphae in the semi-anaerobic environment was observed. Among dimorphic fungi like Candida, the growth environment plays a major role in the morphogenetic behavior of the fungus. The growth conditions described in our study are quite different than that reported by Dumitru et al [23].

The molecular mechanisms involved in the inhibitory effects of liquid isoflurane on *Candida *yeast multiplication and hyphae formation require further exploration. It is possible that cellular shrinkage (Please see Figures 3 and 5) that could lead to apoptosis of yeast cells is a mechanism by which amphotericin B and isoflurane exert antifungal activity.

Previously, it has been reported that volatile anesthetics exert their effects on living cells by one of the three mechanisms: i) altering the lipid bilayer of the plasma membrane, ii) modulation of membrane bound protein function or iii) a combination of both which involves anesthetic interaction with the lipid bilayer altering activity of membrane proteins [24]. Based on previous observations in *Saccharomyces cerevisiae*, it is possible that isoflurane might inhibit nutrient-dependent growth either by affecting membrane permeases or mRNA translation [25,26].

*C. albicans *acts as a silent enemy among healthy individuals and exists mainly by colonizing skin, and mucosal surfaces of oral, digestive tracts and genitalia. Defects in host defense then permit the yeast to promulgate various ailments. Amidst these ailments *Candidal *vaginitis effects almost 80 percent of healthy women once in their lifetime as well as nearly every immunocompromised woman [27,28]. Moreover, maternal Candida vaginitis can transfer infection to newborn infants in the form of thrush and diaper dermatitis [29]. Development of prophylactic measures of the aforementioned situation would have important public health implications for women and infants. Besides superficial infections, Candida-associated systemic infections are also on the rise, particularly among bone marrow transplant patients who have shown higher mortality associated with fungal infections [30]. Although systemic usage of liquid volatile anesthetic via the intravenous route is associated with adverse effects [31], emulsified isoflurane has been developed and intravenous injections of this formulation has been proven to be safe in animal models [32].

Another interesting aspect of this study is *C. albicans *dimorphism which has been extensively studied at both cellular and molecular levels [33-35]. The inhibitory effects of isoflurane on formation of hyphae from various Candidal species will provide the opportunity to study the morphological shift in this opportunistic fungus. Furthermore, currently available robust molecular technologies will assist in identifying genetic elements involved in the dimorphic switch.

Our findings show that liquid isoflurane in culture inhibits yeast multiplication as well as formation of hyphae from *Candida*. It will be essential to further explore the *in vivo *significance of our *in vitro *data in order to translate these findings into clinical application. However, *in vitro *data from our studies strongly suggest that the antifungal activity of liquid isoflurane equals or exceeds Amphotericin B, a gold standard antimycotic currently in use that unfortunately has extremely frequent and serious side effects in humans. The highly lipophilic nature of isoflurane compared to water-soluble Amph B may offer the advantage of better penetration and bioavailability to poorly vascularized tissues. The safety of topically applied liquid isoflurane has been demonstrated in humans [36]. Furthermore, in a canine model, subarachnoid injection of liquid volatile anesthetic agent did not cause permanent motor or sensory neurological injury [37]. Further investigations related to our findings are warranted.

## Materials and methods

### Fungal strain and materials

We used *C. albicans *strain H317, a clinical isolate from the Centers for Disease Control and Prevention (Atlanta, GA), that has been described previously [38]. Isoflurane, USP grade was purchased from Baxter (Baxter Healthcare Corporation, Deerfield, IL).

### Growth media

*C. albicans *H317 cultures were grown in Lee's medium [39] and maintained in 1% yeast extract, 2% peptone and 2% dextrose (YPD) medium. Fresh Lee's medium was prepared bi-weekly and stored at 4°C. An isolated *C. albicans *colony growing on YPD plate was transferred into 20 ml of pre-warmed Lee's medium and allowed to grow at 27°C for initiation of each starter culture.

### Effects of liquid isoflurane on metabolic activity of *C. albicans*

Actively growing *C. albicans *yeast cultures at 27°C were treated with isoflurane in 10 ml sterile plastic syringes (Becton Dickinson, and Company). Briefly, 5.0 ml of fungal suspensions were treated with isoflurane to final concentrations ranging between 0.05% – 0.8% (v/v). Upon addition of isoflurane the syringe plunger and the top cover were tightly closed and shaken for 5 seconds. As a positive control, one of the culture syringes was treated with amphotericin B (4.0 μg/ml), an antifungal agent widely used in clinical practice. Negative control consisted of Candida yeast culture without any treatment. All syringes were tightly capped to create a semi-anaerobic environment and were transferred to 37°C for 16 hours with orbital shaking at 125 rpm. For experiments involving aerobic conditions, the syringe caps were loosened to allow air exchange without risk of external contamination.

Effects of various concentrations of isoflurane and amphotericin B on the metabolic activity of fungi were assessed by volume displaced in the syringes under semi-anaerobic conditions. The volume of displacement from the fungal metabolic activity was recorded with a 3.2 mega pixel digital camera (Figure 1). Upon experiment completion, the optical density (OD) of each culture was also recorded at 600 nm. The effects of various concentrations of isoflurane on the morphology of *C. albicans *were recorded by morphological observations.

### Isoflurane and viability of *C. albicans*

The viability of *C. albicans *upon treatment with isoflurane for 16 hrs in semi-anaerobic and aerobic environments was determined by adding 0.1 ml of culture from the syringes culture in 2 ml of pre-warmed Lee's medium in 15 ml culture tubes. These tubes were shaken on a rotary shaker for further 22 hrs at 37°C in cell-culture tubes. Each culture was thoroughly mixed and 20 μl from each syringe was spread on microscopic slides to capture live images (Olympus inverted microscope with monitor and digital camera, Olympus, Japan). Viability as well as morphological changes were assessed and recorded digitally.

### Statistical methods

All quantitative values are presented as means ± standard error of the mean (SEM). Data comparing fungal metabolism and fungal growth under different conditions were subjected to ANOVA followed by Tukey-Kramer multiple comparisons test. GraphPad statistical software (GraphPad Software, Inc., San Diego, CA) was used to perform the data analysis. Values of p < 0.05 were considered statistically significant.
